# Association between tumor mutations and meningioma recurrence in Grade I/II disease

**DOI:** 10.18632/oncoscience.570

**Published:** 2022-12-09

**Authors:** Jonathan T. Dullea, Vikram Vasan, John W. Rutland, Corey M. Gill, Danielle Chaluts, Daniel Ranti, Ethan Ellis, Varun Subramanium, Annie Arrighi-Allisan, Yayoi Kinoshita, Russell B. McBride, Joshua Bederson, Michael Donovan, Robert Sebra, Melissa Umphlett, Raj K. Shrivastava

**Affiliations:** ^1^Department of Neurosurgery, Icahn School of Medicine at Mount Sinai, New York, NY 10129, USA; ^2^Department of Pathology, Icahn School of Medicine at Mount Sinai, New York, NY 10129, USA; ^3^The Institute for Translational Epidemiology, Icahn School of Medicine at Mount Sinai, New York, NY 10129, USA; ^4^Department of Genetics and Genomic Sciences, Icahn School of Medicine at Mount Sinai, New York, NY 10129, USA; ^5^Sema4, A Mount Sinai Venture, Stamford, CT 06902, USA

**Keywords:** meningioma, molecular genomics, POLE, ATM, CREBBP

## Abstract

Background: Meningiomas are common intracranial tumors with variable prognoses not entirely captured by commonly used classification schemes. We sought to determine the relationship between meningioma mutations and oncologic outcomes using a targeted next-generation sequencing panel.

Materials and Methods: We identified 184 grade I and II meningiomas with both >90 days of post-surgical follow-up and linked targeted next-generation sequencing. For mutated genes in greater than 5% of the sample, we computed progression-free survival Cox-regression models stratified by gene. We then built a multi-gene model by including all gene predictors with a *p*-value of less than 0.20. Starting with that model, we performed backward selection to identify the most predictive factors.

Results: *ATM* (HR = 4.448; 95% CI: 1.517–13.046), *CREBBP* (HR = 2.727; 95% CI = 1.163–6.396), and *POLE* (HR = 0.544; HR = 0.311–0.952) were significantly associated with alterations in disease progression after adjusting for clinical and pathologic factors. In the multi-gene model, only POLE remained a significant predictor of recurrence after adjusting for the same clinical covariates. Backwards selection identified recurrence status, resection extent, and mutations in *ATM* (HR = 7.333; 95% CI = 2.318–23.195) and *POLE* (HR = 0.413; 95% CI = 0.229–0.743) as predictive of recurrence.

Conclusions: Mutations in ATM and CREBBP were associated with accelerated meningioma recurrence, and mutations in POLE were protective of recurrence. Each mutation has potential implications for treatment. The effect of these mutations on oncologic outcomes and as potential targets for intervention warrants future study.

## INTRODUCTION

With an incidence rate of 9.1 per 100,000 person-years, meningiomas are the most common primary central nervous system tumors in the United States [1]. Long-term oncologic outcomes are, in part, predicted by the World Health Organization (WHO) pathologic classification system. Most meningiomas (80–90%) are grade I with a generally favorable disease course. The 5-year recurrence rate for these low-grade tumors was shown to be is 14% when atypical features are absent [2]. Grade II meningiomas, representing 5–15% of these tumors, generally have a worse oncologic course. Aghi et al. demonstrated a 5-year recurrence rate of 41% for these tumors [3]. Though generally considered benign, grade I meningiomas exist that harbor an elevated recurrence risk. This discordance suggests that the WHO score may not fully predict disease outcomes [2]. As such, there is a need to further characterize meningioma disease mechanisms in pursuit of better diagnostics and novel targets to improve treatment paradigms.

Recent work has examined the relationship between meningioma genomics and disease characteristics. These studies have demonstrated that meningiomas are genetically heterogeneous. The gene most frequently altered is the tumor suppressor merlin (*NF2*); a mutation in this gene is present in approximately 45% of meningiomas. Other commonly implicated genes are *TRAF7, AKT1, KLF4, PIKC3A,* and *SMO* [4, 5]. In a study of 553 meningiomas, Yuzawa et al. reported the mutational frequencies at 20%, 9%, 9%, 4.5%, and 3%, for these genes, respectively [6]. Clark et al. described *POLR2A*, *AKT3*, *PRKAR1A*, and *SUFU* as important somatic mutations in meningioma pathogenesis [5, 7]. Prior studies have characterized the effect of these mutations on meningioma grade and tumor location. Presently, the literature has comparatively few reports on the long-term outcomes of meningiomas stratified by genomic alteration. A recent set of studies utilizing the present dataset found that mutation in ARID1A was associated with increased hazard of death and recurrence of primary tumors [8, 9]. Another study also related alterations in DREAM complex transcription with changes in the rate of meningioma recurrence [10]. Another recent study of 121 patients demonstrated that a multi-omic approach can predict recurrence; however, implementation of this technique may be technically challenging in clinical labs due to the cost of the necessary sequencing [11]. Other successful risk stratification methodologies include methylation profiling and analysis of copy number variation [12, 13]. Specifically, loss of chr22q and chr1p is associated with poor prognosis [10]. The recent 2021 update to the WHO classification system has started to include genomic alterations in the determination of grade. Specifically, alterations in *CDKN2A*/*B* and *TERT* promoter result in classification as a WHO grade 3 lesion. With further research, genomic characterization could further improve the prognostic value of post-operative surgical pathology. Future findings could impact post-operative protocols for screening and adjuvant radiotherapy. Lastly, a deeper understanding of genomics could lead to novel targeted adjuvant chemotherapeutics and immunotherapies. In the present study, we aimed to further explore the genomic underpinnings of meningioma recurrence.

## RESULTS

### Study cohort characteristics

There were a total of 184 meningiomas that fit inclusion criteria in the tissue bank. The median age of participants is 60.4 years old, and there were a greater number of women than men in the cohort. 58.7% of the tumors were gross-totally resected. There was an intentional overabundance of grade 2 disease present in the cohort. The clinical characteristics of the study cohort are shown in Table 1.

**Table 1:** Clinical characteristics of the cohort

	Value
**Age at surgery**	
Mean (SD)	58.4 (13.4)
Median (Min, Max)	60.4 (23.1, 85.1)
**Sex**	
Female	120 (65.2%)
Male	64 (34.8%)
**Resection extent**	
Gross-total Resection	108 (58.7%)
Sub-total Resection	65 (35.3%)
	11 (6.0%)
**Recurrence status**	
Primary	127 (69.0%)
Recurrent	57 (31.0%)
**Grade**	
Grade 1	50 (27.2%)
Grade 2	134 (72.8%)

### Genomic sequencing

**Figure 1:**
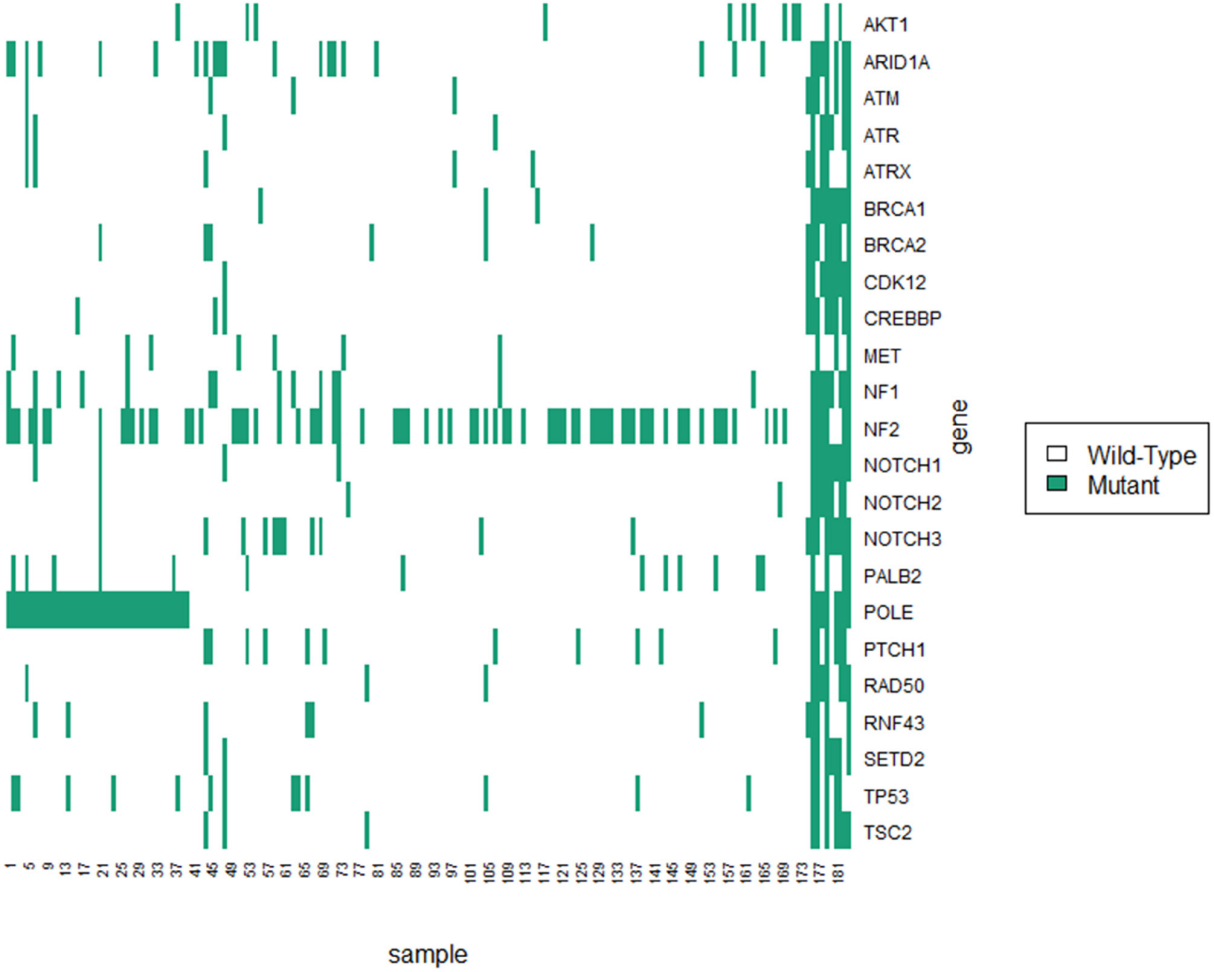
Mutational landscape of samples included in this study.

Sequencing data from this cohort revealed substantial mutational heterogeneity. The most prevalent gene alteration in the cohort of 184 patients was a mutation in NF2. This gene was altered in 79 (42.9%) of tumors. The second most prevalent mutation was in *POLE,* a DNA polymerase (26.1%). The mutational distribution of the most prevalent mutations is shown in Figure 1.

### Survival analysis

Analysis of clinical features alone demonstrated a statistically significant 167% increase in the hazard of recurrence for previously recurrent tumors (HR (95% CI) = 2.67 (1.60–4.45), *p* = 0.0002). Further, sub-totally resected tumors had a 74% increase in the hazard of recurrence (HR (95% CI) = 1.73 (1.04–2.90), *p* = 0.034). A one-year increase in age at surgery was not associated with a significant increase in hazard of recurrence. (HR (95% CI) = 1.02 (1.00– 1.04), *p* = 0.0608). There was also no significant relationship between increased tumor grade and recurrence status (HR (95% CI) = 1.67 (0.96–2.92), *p* = 0.072). Further, there was no significant relationship between sex and tumor recurrence (Table 2).

**Table 2:** Cox regression results for clinical variable only mode

Clinical characteristic	Hazard ratio (95% CI)	*p*-value
Age (years)	1.019 (0.999, 1.04)	0.0608
Sex (Male)	1.31 (0.788, 2.177)	0.298
Grade		0.0723
Grade 1	1.000 (referent)	
Grade 2	1.669 (0.955, 2.918)	
Recurrence Status		0.0002
Primary	1.000 (referent)	
Recurrent	2.67 (1.601, 4.454)	
Resection Extent		0.0343
Gross-total Resection	1.000 (referent)	
Sub-total Resection	1.737 (1.042, 2.897)	

**Figure 2:**
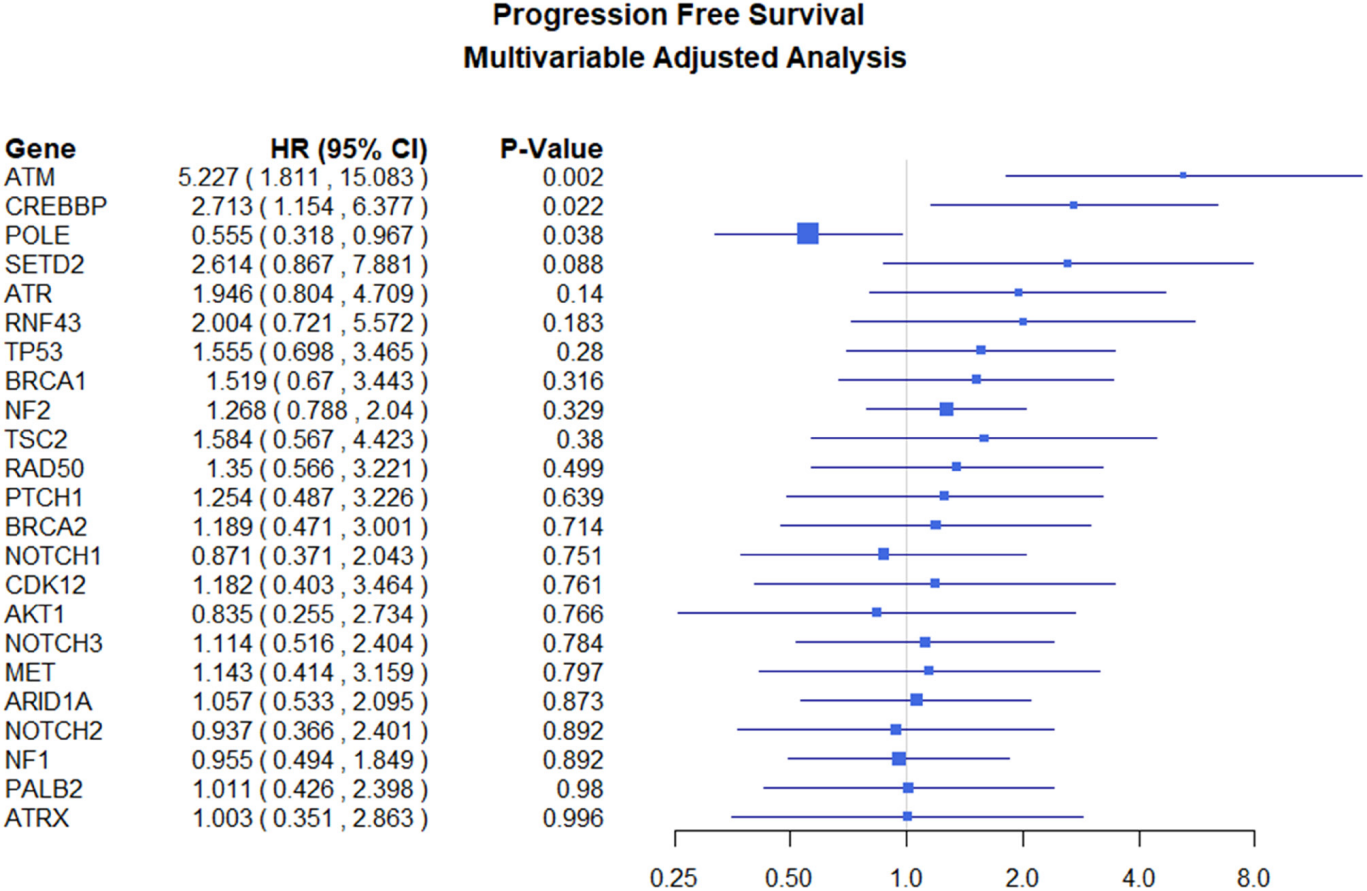
Adjusted progression-free survival hazard ratios for genes examined.

All genomic survival analysis results are summarized in Table 3. The unadjusted analyses did not demonstrate any genes significantly associated with PFS. After adjusting for grade, recurrence status, resection extent, and age there are statistically significant associations between genomic alterations and progression-free survival. *ATM* is associated with a 348% increase in the hazard of progression (HR (95% CI) = 4.448 (1.517, 13.05)). *CREBBP* was also found to predict a greater hazard of recurrence (HR (95% CI) = 2.73 (1.163, 6.396)). Conversely, alterations in *POLE* are associated with a significant protective effect (HR (95% CI) = 0.544 (0.311, 0.952)). The results of this analysis are shown in Figure 2.

**Table 3:** Survival analysis modeling results

Gene		Model							
		Unadjusted		Uni-genomic adjusted^§^		Multi-genomic adjusted^§^		Variable selected^§§^	
Name	*N*	HR (95% CI)	*p*-value	HR (95% CI)	*p*-value	HR (95% CI)	*p*-value	HR (95% CI)	*p*-value
AKT1	12	0.51 (0.16, 1.63)	0.254	0.895 (0.273, 2.933)	0.8542	**–**	**–**	**–**	**–**
ARID1A	27	1.22 (0.71, 2.1)	0.462	1.101 (0.561, 2.161)	0.7798	**–**	**–**	**–**	**–**
ATM	11	1.67 (0.8, 3.48)	0.172	4.448 (1.517, 13.046)	**0.0066**	3.323 (0.72, 15.342)	0.1239	7.333 (2.318, 23.195)	0.0007
ATR	10	1.81 (0.87, 3.78)	0.113	1.953 (0.802, 4.754)	0.1403	2.152 (0.824, 5.616)	0.1175	**–**	**–**
ATRX	10	1.01 (0.46, 2.22)	0.977	1.062 (0.369, 3.057)	0.9117	**–**	**–**	**–**	**–**
BRCA1	12	1.18 (0.57, 2.47)	0.656	1.509 (0.668, 3.407)	0.3225	**–**	**–**	**–**	**–**
BRCA2	14	1.09 (0.52, 2.27)	0.825	1.445 (0.564, 3.7)	0.443	**–**	**–**	**–**	**–**
CDK12	10	0.99 (0.43, 2.3)	0.982	1.28 (0.433, 3.781)	0.6548	**–**	**–**	**–**	**–**
CREBBP	11	1.8 (0.87, 3.75)	0.114	2.727 (1.163, 6.396)	**0.0211**	2.218 (0.669, 7.35)	0.1925	**–**	**–**
MET	10	0.91 (0.33, 2.5)	0.856	0.996 (0.358, 2.769)	0.9939	**–**	**–**	**–**	**–**
NF1	22	1.09 (0.61, 1.94)	0.781	0.97 (0.5, 1.883)	0.9283	**–**	**–**	**–**	**–**
NF2	79	1.18 (0.76, 1.82)	0.453	1.166 (0.724, 1.879)	0.5275	**–**	**–**	**–**	**–**
NOTCH1	13	0.95 (0.46, 1.99)	0.9	0.97 (0.41, 2.299)	0.9453	**–**	**–**	**–**	**–**
NOTCH2	10	0.87 (0.38, 2.03)	0.754	1.06 (0.408, 2.756)	0.9043	**–**	**–**	**–**	**–**
NOTCH3	20	0.79 (0.4, 1.54)	0.483	1.263 (0.576, 2.768)	0.5601	**–**	**–**	**–**	**–**
PALB2	17	0.72 (0.33, 1.57)	0.412	1.017 (0.428, 2.415)	0.9701	**–**	**–**	**–**	**–**
POLE	48	0.68 (0.41, 1.12)	0.128	0.544 (0.311, 0.952)	**0.0328**	0.386 (0.207, 0.719)	**0.0027**	0.413 (0.229, 0.743)	**0.0032**
PTCH1	17	0.92 (0.45, 1.85)	0.81	1.543 (0.594, 4.007)	0.3729	**–**	**–**	**–**	**–**
RAD50	10	1.23 (0.56, 2.7)	0.602	1.256 (0.526, 2.999)	0.6074	**–**	**–**	**–**	**–**
RNF43	11	1.26 (0.61, 2.63)	0.535	1.929 (0.693, 5.367)	0.2082	**–**	**–**	**–**	**–**
TP53	18	1.21 (0.65, 2.24)	0.556	1.54 (0.693, 3.421)	0.2887	**–**	**–**	**–**	**–**
TSC2	10	1.16 (0.53, 2.55)	0.707	1.618 (0.583, 4.491)	0.3559	**–**	**–**	**–**	**–**

^§^Adjusted for Grade, Recurrence Status, and Resection Extent. Samples with missing data were excluded (total sample size = 173). ^§§^Adjusted for Recurrence Status, and Resection Extent after variable selection. Samples with missing data were excluded (total sample size = 173). 4.448 (1.517, 13.046), 2.727 (1.163, 6.396), 0.544 (0.311, 0.952). 0.386 (0.207, 0.719). 7.333 (2.318, 23.195), 0.413 (0.229, 0.743).

**Figure 3:**
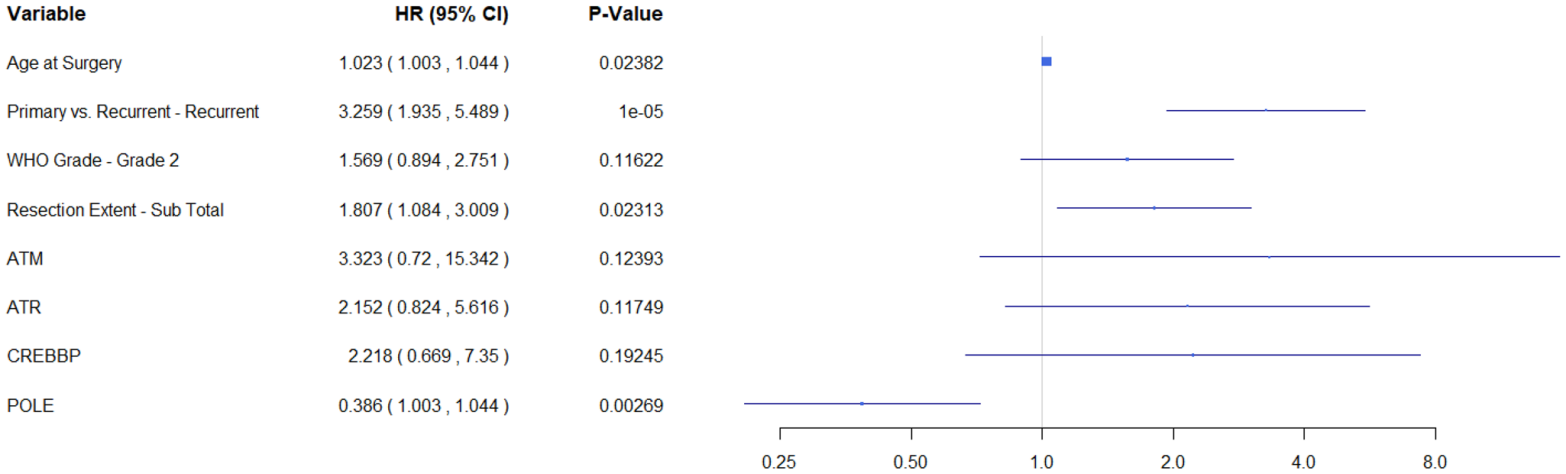
Multi-gene model demonstrates that POLE is predictive of improved survival.

**Figure 4:**
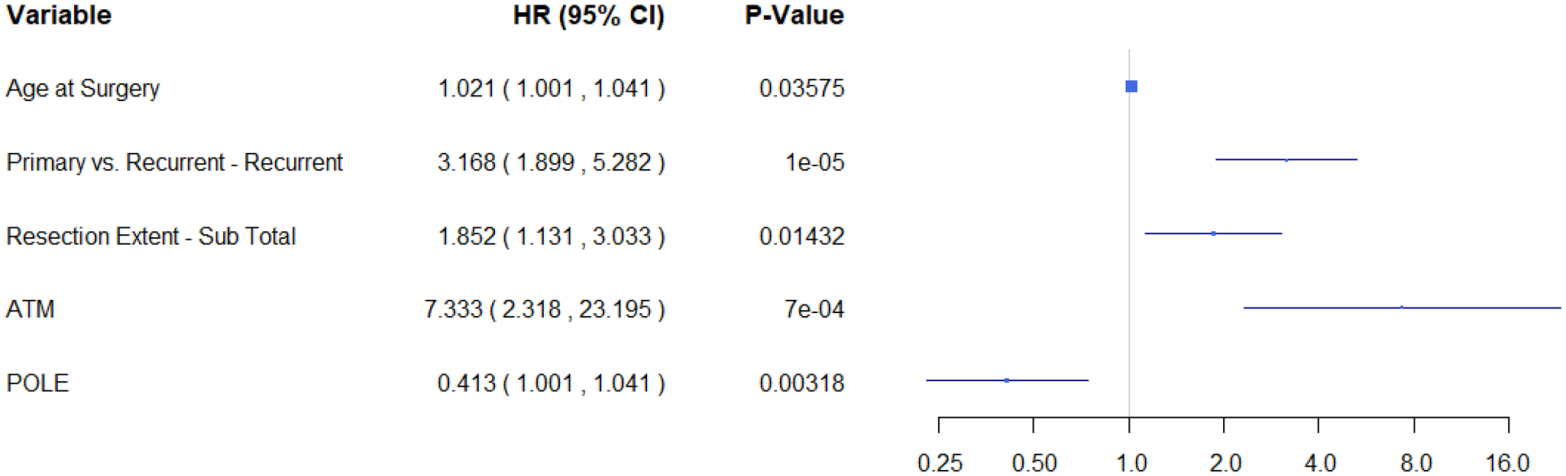
Variable selected model demonstrates that POLE and ATM are predictive of recurrence.

Using the findings from the uni-genomic adjusted models, a composite model was built from the genes most significantly associated with progression-free survival (*ATM*, *ATR*, *CREBBP*, and *POLE*). After adjusting for all the clinical covariates and other potentially predictive genes, only alterations in *POLE* remained predictive of recurrence (HR (95% CI) = 0.386 (0.207, 0.719)). (Table 3 and Figure 3). Using this model as a starting point, backward variable selection was utilized. The variables found to be most predictive of recurrence were recurrence status, resection extent, *ATM*, and *POLE*. (Table 3 and Figure 4) Interestingly, WHO grade was not found to improve the predictive accuracy of the model. *ATM* was again detrimental (HR (95% CI) = 7.333 (2.32, 23.20)) and *POLE* was protective (HR (95% CI) = 0.413 (0.23, 0.74)). These results are also summarized in Table 3. Note that variance inflation factors (VIF) were computed to assess for collinearity of the final model. Max VIF for included predictors was 1.26 indicating no concern for collinearity.

**Figure 5:**
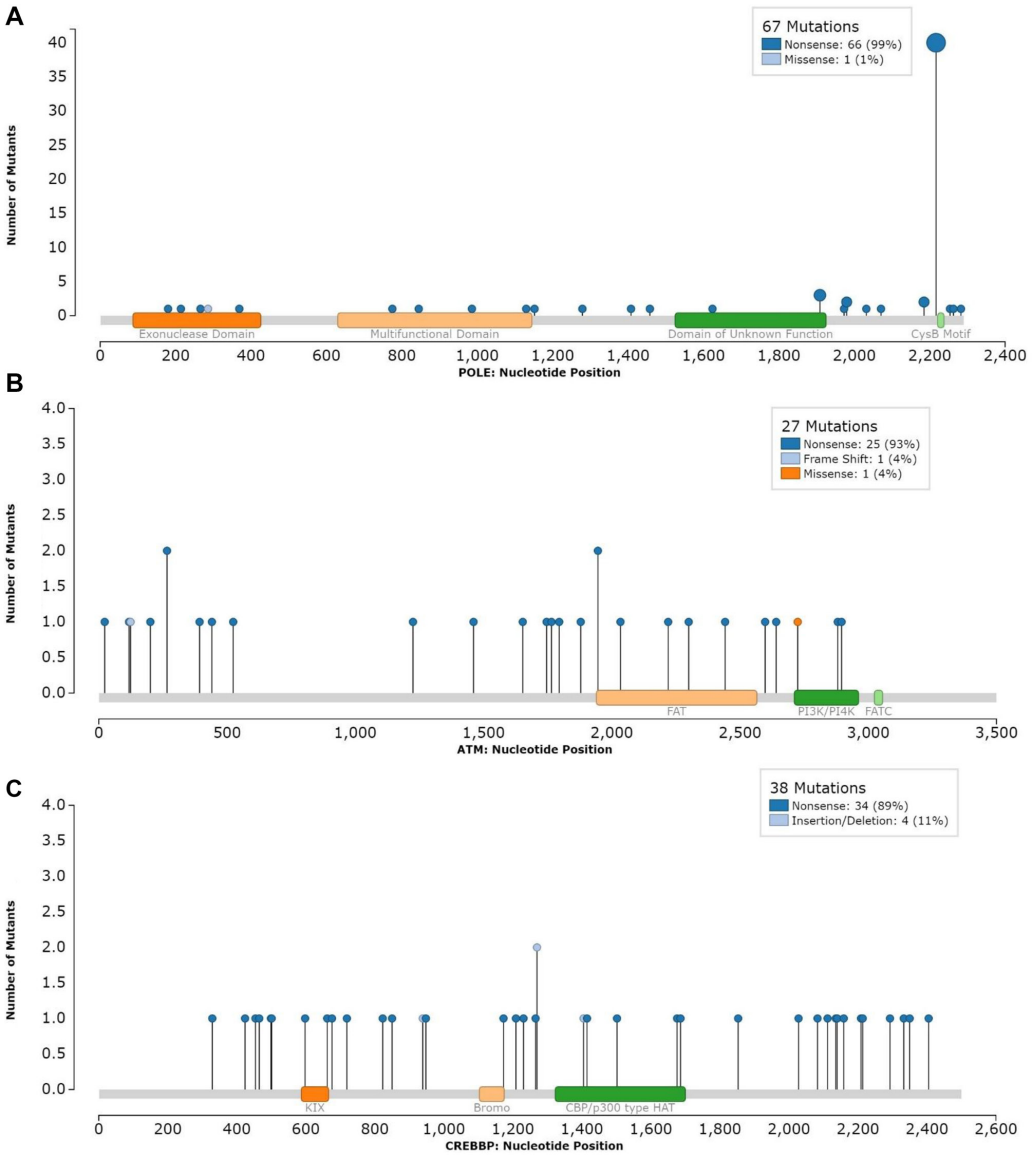
Mutations in (**A**) POLE, (**B**) ATM, (**C**) CREBBP.

To determine the effect of interaction between recurrent status and the identified genes of interest with time to recurrence, we performed survival analysis stratified by recurrence status. The same patterns of increased hazard of recurrence of *ATM* and *CREBBP* remained consistent in the primary tumors. Similarly, *ATM* was found to portend worse outcome in recurrent tumors. There was, however, no longer a significant association between *CREBBP* and recurrence in the already recurrent tumors; however, there were only 4 samples which may have influenced this result. With regards to *POLE*, we demonstrated that mutations in *POLE* were most protected in recurrent tumors. The relationship was somewhat attenuated in primary tumors to the point resulting in a statistically insignificant result. All subgroup results are shown in Table 4. The mutational characteristics of *POLE*, *ATM*, and *CREBBP* across the study are shown in Figure 5 and Supplementary Table 1.

**Table 4:** Progression-free survival results stratified by recurrent status for *ATM, CREBBP, POLE,* and *NF2* mutant tumors

Gene name	*N*	Uni-genomic adjusted^§^	
		HR (95% CI)	*p*-value
Primary tumors (*n* = 120)			
*ATM*	7	3.54 (0.93–13.49)	0.064
*CREBBP*	7	3.08 (0.85–11.21)	0.088
*POLE*	28	0.88 (0.42–1.95)	0.758
*NF2*	53	1.04 (0.48–2.24)	0.927
Recurrent tumors (*n* = 54)			
*ATM*	4	6.62 (0.76–57.81)	0.087
*CREBBP*	4	1.80 (0.53–6.11)	0.345
*POLE*	20	0.54 (0.25–1.14)	0.106
*NF2*	26	1.03 (0.50–2.15)	0.926

^**§**^Adjusted for grade, recurrence status, and resection extent. Samples with missing data were excluded.

## DISCUSSION

Recent studies have demonstrated that insight into meningioma pathogenesis and prognosis lies, in part, in understanding the genomic underpinnings of disease. By exploring the molecular basis of outcomes, researchers have begun to characterize the factors that protect from and drive the recurrence of disease after resection. In the present study, we found that mutations in three genes, *ATM*, *POLE*, and *CREBBP*, were associated with changes in time to disease recurrence after resection. Similar patterns were noted in cohorts restricted to a single prior recurrence status. We found relationships suggesting that *ATM* predictive of recurrence in both primary and recurrent tumors. Additionally, we found evidence suggesting that *POLE* is associated with improved survival in recurrent tumors. Finally, our data suggests that *CREBBP* is most deleterious in primary tumors. The loss of significance in the sub-group analysis was likely in part due to sample size restriction. Overall, we found that the most predictive factors in determining recurrence were age at surgery, prior history of recurrence, resection extent, and mutations in *ATM*, *POLE,* and *CREBBP*. Interestingly, some known drivers of meningioma pathogenesis, such as TP53 and NF2, were not related to increased hazard of recurrence; this is likely a result of no increased risk over the baseline for all meningiomas. Another possibility is that the study was underpowered to detect increased hazard for lower prevalence alterations.

*ATM* is the gene implicated in the development of ataxia-telangiectasia, a disease clinically characterized by ataxia, immunosuppression, and predisposition to malignancies. For patients with this autosomal recessive disease, these symptoms arise from dysfunction of this kinase typically activated under conditions of double-stranded DNA breaks. Somatic mutations in this gene have been found in many solid and hematologic cancers [14]. In individuals with B-cell lymphoma, the presence of a mutation in ATM is associated with a worse prognosis. Prior studies have demonstrated that there is a relationship between meningiomas and mutations in *ATM*. First, a specific germline *ATM* haplotype is over-represented in patients with meningiomas [15]. Another study demonstrated that *ATM* mutant Grade II meningiomas display poor long-term outcomes [16]. To date, the present study is the largest to report on the association of *ATM* and elevated risk of meningioma recurrence. Most of the ATM mutations demonstrated here are nonsense mutations, as are seen in ataxia telangiectasia, that likely lead to a loss of function. This finding may have relevance to the development of future treatment paradigms. Given the resultant faulty dsDNA break repair process, tumors with altered *ATM* may be more sensitive to agents that lead to dsDNA breaks (ionizing radiation and chemotherapeutics) [14, 17]. Further study is warranted to determine if ATM mutant meningiomas would garner an outsized survival benefit from treatment with such an agent or radiation protocol.

Our analysis also demonstrated that mutations in *POLE* are associated with an 45% reduction in the hazard of recurrence. *POLE* is a DNA polymerase that is involved in replication and repair [18]. There is a precedent of a protective effect of mutations in *POLE* in endometrial cancer [19]. In endometrial cancer, a n-terminal exonuclease mutation in *POLE* leads to a hypermutant phenotype. This phenotype leads to improved survival through immunological control of the resulting immunogenic phenotype [19, 20]. The mechanism in meningiomas is likely distinct from the mechanism in the other malignancies. As shown in Figure 1, many of the *POLE* mutant samples were not exclusively part of the exceptionally high mutational burden cluster. In recently published work using this dataset, we demonstrated that many of these mutations are outside of the previously implicated exonuclease domain [21]. Instead, the mutations were predominantly present in a c-terminal domain important for polymerase stabilization [21]. The c-terminal mutations were associated with a modest increase in mutational burden. In that work, we hypothesized that this increase was related to preferential activation of lower fidelity polymerases due to *POLE* complex destabilization. That study also demonstrated an increase in CD8+ infiltration for the tumors with a mutation in POLE. We further hypothesized that the slightly elevated mutational burden may lead to greater immunological control and thus better oncologic outcomes.

The present study builds on the characterization of POLE as a potentially protective mutation in meningiomas. We herein demonstrated that POLE mutations are among the most important predictors of outcomes of the genes analyzed in this cohort. We further demonstrated evidence that this may result mainly from an effect in recurrent tumors. After considering a subset of potentially predictive genes, the automated variable selection process identified that the inclusion of *POLE* in the model improved the prediction of progression-free survival time. This finding may inform the development of future therapeutics. Immunotherapies have found utility in treating other *POLE* mutant malignancies. The result holds in patients without microsatellite instability [22]. It remains to be seen if *POLE* mutant meningiomas would garner the same beneficial outcomes from checkpoint inhibitors. If a future study again demonstrates this relationship, initiation of a randomized trial of checkpoint inhibitors in well-selected, potentially immunogenic, meningiomas may be warranted.

The last gene found to be potentially predictive of alterations in survival time was *CREBBP*, a chromatin remodeling gene. We found that mutations in this gene were associated with a significant increase in the hazard of recurrence. *CREBBP* is the gene mutated in Rubinstein-Taybi syndrome, an autosomal dominant syndrome characterized by intellectual disability and increased risk of benign tumor formation, including meningiomas [20]. Alterations in this are implicated in many solid malignancies, including small cell lung cancer [23]. Additionally, brain metastases of lung adenocarcinoma with mutation in *CREBBP* have been shown to be associated with poorer prognosis compared to wild-type [24]. With regards to meningiomas, a recent study identified *CREBBP* as present in aggressive meningiomas [11]. In previously reported results from this cohort, alterations in ARID1A, another chromatin-remodeling-related gene, were associated with an increased hazard of death and recurrence of primary tumors [8, 9]. The present finding, in the context of the prior work, indicates that there may be a relationship between chromatin remodeling dysfunction and meningioma outcomes. The present study is the largest to report directly on *CREBBP* as a gene associated with tumor prognosis. Herein we demonstrated that nonsense mutations, which are likely loss of function mutations, in CREBBP are associated with worse outcomes. This finding, too, could be relevant to the development of future treatment modalities. Recent *in-vitro* and animal model work in small cell lung cancer has demonstrated that *CREBBP* mutant tumors may be preferentially sensitive to HDAC1 inhibitors [23, 25]. Further validation in meningiomas could lead to HDAC inhibition as a possible adjuvant treatment to prevent a recurrence in well-selected tumors.

A final finding from this study is that inclusion of the WHO grade did not improve the prediction of time to recurrence among grade I and II tumors. The automated selection process omitted this as a predictor. This result provides evidence that in stratifying the risk of recurrence between grade I and II disease, other factors such as recurrence status and resection extent are more informative than grade. Additionally, this provides further evidence that the classification of meningiomas should include additional genomic elements. This model only included grade I–II disease; it is likely that WHO grade would remain predictive in a dataset with grade III tumors.

### Strengths and limitations

This genomics study allowed us to link various genes with follow-up data to find associations between mutations and the clinical course of patients. A major strength of this study is that we used clinically tractable genomic techniques that, if further validated, could be helpful for clinical decision making. Of note, we did not find statistical significance from the *NF2* mutation, which has been highlighted in a recent impactful study [16]. This same study used a more encompassing multi-omic approach, introducing other factors surrounding meningioma prognosis, like DNA methylation and RNA sequencing. Despite the discrepancy in *NF2* mutation and less comprehensive approach, we still believe our results about other potential driver mutations offer some insights into treatment. Another limitation is that our dataset, although among the largest meningioma cohorts in existence, is still somewhat small compared to ones that exist for other tumors. The sample size partly limits the statistical power of our results for more subtle findings. Given that this study is exploratory we aimed to identify genes potentially related to meningioma clinical outcomes. We therefore did not perform the analysis under a strict statistical framework accounting for multiple comparisons such as one which adhered to the Bonferroni correction. This is justified given that the mutational landscape of meningiomas remains presently under characterized. As such, we sought to limit the chance of a type II error at the expense of a higher chance of a type I error. Given this limitation, the data herein presented requires future validation in an independent dataset. Another limitation is that our study does not include grade III disease. We excluded grade III because there were issues with the proportional hazards assumption necessary for Cox-regression. This necessary exclusion limited the generalizability of the results. Also, as previously mentioned, another limitation is that the targeted sequencing panel does not exhaustively cover meningioma driver mutations. Lastly, we do not explore copy number variation in this study, but it is planned for future work.

## MATERIALS AND METHODS

### Study cohort

The institutional review board at the senior author’s institution reviewed this study. The study was verified to follow all ethical guidelines laid out in the 1964 Declaration of Helsinki and the later amendments and the Health Insurance Portability and Accountability Act of 1996 (HIPAA) guidelines. Due to the retrospective nature of this study, informed consent was waived. Meningiomas with available formalin-fixed paraffin-embedded (FFPE) tissue from 255 resections performed between 2001 to 2018 were selected for inclusion. A board-certified neuropathologist reviewed histopathological diagnosis, grade, and purity of each case according to 2016 World Health Organization (WHO) guidelines. We selected those with WHO grade I and II tumors from this cohort of patients and at least 90 days of post-operative follow-up in our analysis cohort. Patients with limited follow-up were excluded to ensure that the post-operatively detected tumor was a true recurrence and not residual unresected tumor. We collected clinical characteristics and recurrence outcomes for the patients included through a retrospective review of the medical record. To model progression-free survival (PFS), we defined follow-up time from surgery to recurrence detected on radiographic imaging. Loss to follow-up and death were defined as censoring events. Study follow-up continued through May 2020.

### Targeted next-generation sequencing

We performed next-generation targeted sequencing using a commercially available platform that covers 143 genes broadly implicated in human malignancy for all the samples that met the inclusion criteria defined above. First, DNA was extracted from the FFPE tissue using Maxwell FFPE Plus DNA Purification Kit (Promega, Madison, WI, USA). Using a previously described method, we generated DNA libraries using AmpliSeq Oncomine Comprehensive research panel version 3.0 (ThermoFisher Scientific, Waltham, MA, USA) [26]. Analysis of the sequencing data was performed using Torrent Suite (version 5.6.0 and 5.0.8) (ThermoFisher Scientific, Waltham, MA, USA) and Ion Reporter (version 5.2, 5.6, and 5.8) (ThermoFisher Scientific, Waltham, MA, USA). We selected this panel due to its coverage of 161 genes broadly implicated in human malignancy and meningioma development. Notably, this panel has coverage of the genes *NF2*, *AKT1*, *SMO*, and *PI3KCA,* which are all implicated in the pathogenesis of meningiomas. Unfortunately, this commercial sequencing panel does not have coverage of several other genes involved as meningioma driver mutations (*KLF4*, *TRAF7*, or *POLR2A*). We considered the effect of mutations present in at least 5% of the cohort in this analysis. We visualized the k-modes clustered mutational landscape of the meningiomas with a heatmap. For mutations of interest, the specific alterations were visualized with the R package “mutsneedle” [27]. This cohort, and the associated sequencing data, have been previously reported [8, 9, 28–32].

### Statistical methods

Cox proportional hazards models were employed to model progression-free survival as defined above. A model with just pathological clinical and pathological features was built first. Covariates with *p*-value less than 0.20 were included in further analysis, where stated. Univariable models were then built only adjusting for genomic alterations in the genes with mutations present in at least 5% of the cohort. Next, adjusted models were built for each gene adjusted for the identified clinical and pathological covariates. Next, we built a multi-genomic model utilizing the same clinical covariates as above and all genes with an adjusted *p*-value of less than 0.20. Lastly, we built an optimized model by performing automatic backward selection with *p*-values as the selection criterion and with the multi-genomic model as a starting point. All statistical tests were performed with a significance level of 0.05 in the R programing language (version 4.0.4).

## CONCLUSIONS

Though meningioma patients often see favorable oncological outcomes, many tumors still recur post-surgically. There is a dearth of literature surrounding the genomic factors that are associated with recurrence. Researchers have recently begun to characterize multi-omics disease prediction tools; however, it may be some time before these see wide clinical utility. We aimed to study multiple mutated genes using an inexpensive, commercially available tool. With this, we explored the impact of these mutations on recurrence-free survival time. We found that *ATM, POLE,* and *CREBBP* had a significant effect on disease progression post-surgically. *ATM* and *CREBBP* were associated with a higher risk of recurrence, while *POLE* was associated with a lower risk of recurrence, thus potentially serving as a protective factor. Further characterization of these genes could become helpful in the development of future treatment paradigms for meningiomas.
